# Why Public Health Agencies Cannot Depend on Good Laboratory Practices as a Criterion for Selecting Data: The Case of Bisphenol A

**DOI:** 10.1289/ehp.0800173

**Published:** 2008-10-22

**Authors:** John Peterson Myers, Frederick S. vom Saal, Benson T. Akingbemi, Koji Arizono, Scott Belcher, Theo Colborn, Ibrahim Chahoud, D. Andrew Crain, Francesca Farabollini, Louis J. Guillette, Terry Hassold, Shuk-mei Ho, Patricia A. Hunt, Taisen Iguchi, Susan Jobling, Jun Kanno, Hans Laufer, Michele Marcus, John A. McLachlan, Angel Nadal, Jörg Oehlmann, Nicolás Olea, Paola Palanza, Stefano Parmigiani, Beverly S. Rubin, Gilbert Schoenfelder, Carlos Sonnenschein, Ana M. Soto, Chris E. Talsness, Julia A. Taylor, Laura N. Vandenberg, John G. Vandenbergh, Sarah Vogel, Cheryl S. Watson, Wade V. Welshons, R. Thomas Zoeller

**Affiliations:** 1 Environmental Health Sciences, Charlottesville, Virginia, USA; 2 Division of Biological Sciences, University of Missouri, Columbia, Missouri, USA; 3 Department of Anatomy, Physiology and Pharmacology, College of Veterinary Medicine, Auburn University, Auburn, Alabama, USA; 4 Faculty of Environmental and Symbiotic Science, Prefectural University of Kumamoto, Tsukide, Kumamoto, Japan; 5 Department of Pharmacology and Cell Biophysics, Center for Environmental Genetics, University of Cincinnati, Cincinnati, Ohio, USA; 6 The Endocrine Disruption Exchange, Paonia, Colorado, USA; 7 Institut für Klinische Pharmakologie und Toxikologie Charité, Universitätsmedizin Berlin, Campus Benjamin Franklin, Berlin, Germany; 8 Department of Biology, Maryville College, Maryville, Tennessee, USA; 9 Dipartimento di Fisiologia, Università di Siena, Siena, Italy; 10 Department of Zoology, University of Florida, Gainesville, Florida, USA; 11 School of Molecular Biosciences, Washington State University, Pullman, Washington, USA; 12 Department of Environmental Health, University of Cincinnati, Cincinnati, Ohio, USA; 13 National Institutes of Natural Science, Okazaki Institute for Integrative Bioscience, Bioenvironmental Science, Okazaki, Japan; 14 Department of Biological Sciences, Brunel University, Uxbridge, United Kingdom; 15 Division of Cellular and Molecular Toxicology, National Institute of Health Sciences, Tokyo, Japan; 16 Department of Molecular and Cell Biology, University of Connecticut, Storrs, Connecticut, USA; 17 Department of Epidemiology, Rollins School of Public Health, Emory University, Atlanta, Georgia, USA; 18 Center for Bioenvironmental Research, Tulane and Xavier Universities, New Orleans, Louisiana, USA; 19 Instituto de Bioingeniería and CIBERDEM, Universidad Miguel Hernández de Elche, Alicante, Spain; 20 Goethe University Frankfurt am Main, Department Aquatic Ecotoxicology, Frankfurt, Germany; 21 Hospital Clínico, CIBERESP, University of Granada, Granada, Spain; 22 Dipartimento di Biologia Evolutiva e Funzionale, Universita’ di Parma, Parma, Italy; 23 Tufts Medical School, Boston, Massachusetts, USA; 24 Institute of Pharmacology and Toxicology, University of Wuerzburg, Wuerzburg Germany; 25 Charité University Medical School Berlin, Berlin, Germany; 26 Department of Biology, North Carolina State University, Raleigh, North Carolina, USA; 27 Chemical Heritage Foundation, Philadelphia, Pennsylvania, USA; 28 Biochemistry and Molecular Biology, University of Texas Medical Branch, Galveston, Texas, USA; 29 Department of Biomedical Sciences, University of Missouri, Columbia, Missouri, USA; 30 Biology Department, University of Massachusetts, Amherst, Massachusetts, USA

**Keywords:** bisphenol A, endocrine disruptors, FDA, Food and Drug Administration, GLP, good laboratory practices, low-dose, nonmonotonic, positive control

## Abstract

**Background:**

In their safety evaluations of bisphenol A (BPA), the U.S. Food and Drug Administration (FDA) and a counterpart in Europe, the European Food Safety Authority (EFSA), have given special prominence to two industry-funded studies that adhered to standards defined by Good Laboratory Practices (GLP). These same agencies have given much less weight in risk assessments to a large number of independently replicated non-GLP studies conducted with government funding by the leading experts in various fields of science from around the world.

**Objectives:**

We reviewed differences between industry-funded GLP studies of BPA conducted by commercial laboratories for regulatory purposes and non-GLP studies conducted in academic and government laboratories to identify hazards and molecular mechanisms mediating adverse effects. We examined the methods and results in the GLP studies that were pivotal in the draft decision of the U.S. FDA declaring BPA safe in relation to findings from studies that were competitive for U.S. National Institutes of Health (NIH) funding, peer-reviewed for publication in leading journals, subject to independent replication, but rejected by the U.S. FDA for regulatory purposes.

**Discussion:**

Although the U.S. FDA and EFSA have deemed two industry-funded GLP studies of BPA to be superior to hundreds of studies funded by the U.S. NIH and NIH counterparts in other countries, the GLP studies on which the agencies based their decisions have serious conceptual and methodologic flaws. In addition, the U.S. FDA and EFSA have mistakenly assumed that GLP yields valid and reliable scientific findings (i.e., “good science”). Their rationale for favoring GLP studies over hundreds of publically funded studies ignores the central factor in determining the reliability and validity of scientific findings, namely, independent replication, and use of the most appropriate and sensitive state-of-the-art assays, neither of which is an expectation of industry-funded GLP research.

**Conclusions:**

Public health decisions should be based on studies using appropriate protocols with appropriate controls and the most sensitive assays, not GLP. Relevant NIH-funded research using state-of-the-art techniques should play a prominent role in safety evaluations of chemicals.

Regulatory agencies in the United States and the European Union (EU) have justified the decision to declare the estrogenic chemical bisphenol A (BPA) safe at current levels of human exposure based on a few studies conducted using Good Laboratory Practices (GLP). In contrast, these agencies have rejected for consideration in their risk assessment of BPA hundreds of laboratory animal and mechanistic cell culture studies conducted by academic and government scientists reporting harm at very low doses of BPA. These studies were rejected primarily because they were not conducted using GLP. We suggest that decisions based on this logic are misguided and will result in continued risk to public health from exposure to BPA, as well as other manmade chemicals.

GLP is a federal rule for conducting research on the health effects or safety testing of drugs or chemicals submitted by private research companies for regulatory purposes. The GLP outlines basic guidelines for conducting scientific research, including the care and feeding of laboratory animals, standards for facility maintenance, calibration and care of equipment, personnel requirements, inspections, study protocols, and collection and storage of raw data (Goldman 1988). These regulations were developed in response to widespread misconduct by private research companies; this misconduct was possible because their data usually do not go through the rigorous, multi stage scientific review that is normal for academic data funded by federal agencies and published in the peer-reviewed literature. The lack of these safeguards from academic science had enabled fraud. The U.S. Food and Drug Administration (U.S. FDA) first issued rules for GLP in 1978 after a 2-year federal investigation into sloppy laboratory practices of a number of private research companies (Lublin 1978; Markowitz and Rosner 2002). What began as serious concerns about poor quality research expanded into a criminal investigation of Industrial Bio-Test (IBT), one of the largest private laboratories at the time and a subsidiary of Nalco Chemical Company. In response to the federal investigation, the U.S. Environmental Protection Agency (EPA) demanded that 235 chemical companies reexamine the > 4,000 tests conducted by the laboratory. In 1983, three men from IBT were found guilty of deliberating doctoring data and were sentenced to prison (Lublin 1978; Markowitz and Rosner 2002). The fraudulent practices of IBT brought into question 15% of the pesticides approved for use in the United States. That same year, the U.S. EPA issued similar GLP rules for regulatory testing.

Both the U.S. FDA (2008a) and European Food Safety Authority (ESFA 2006) have recently published documents demonstrating that their decision to continue to declare BPA safe at current exposure levels was based primarily on the results of a few industry-funded studies that followed GLP guidelines. These decisions stand in stark contrast to the decisions concerning the potential risks to human health reached by a panel of 38 experts at a U.S. National Institutes of Health (NIH)-sponsored conference, who published The Chapel Hill Consensus Statement (vom Saal et al. 2007), as well as five review articles (Crain et al. 2007; Keri et al. 2007; Richter et al. 2007a; Vandenberg et al. 2007a; Wetherill et al. 2007). These peer-reviewed articles covered approximately 700 articles concerning BPA and represented a comprehensive review of the literature as of the end of 2006. In addition, the U.S. FDA draft decision contradicted the conclusions reached by the National Toxicology Program (NTP), which had spent 2 years investigating this question (NTP 2008). An important role of the NTP is to advise the U.S. FDA about the science relating to toxic chemicals in food, but in an unusual move, the U.S. FDA chose to release its draft report before the release of the final report on BPA by the NTP and without indicating who at the U.S. FDA was involved in preparing the draft report (U.S. FDA 2008b). At a hearing on 16 September 2008 regarding the draft report on BPA, the U.S. FDA announced that their goal was to have a subcommittee of the U.S. FDA Science Board complete a review of the draft decision by the end of October 2008. This would presumably also involve review by the subcommittee members of the approximately 1,000 articles relating to BPA.

We believe that the methods employed in chemical industry–sponsored GLP studies are incapable of detecting low-dose endocrine- disrupting effects of BPA and other hormonally active chemicals. Detecting endocrine-disrupting effects at low doses of chemicals such as BPA requires sophisticated and modern assays and analyses that have been developed in advanced, usually federally funded laboratories over the past decade. This is especially apparent when one examines what is now known about functional effects of BPA on a wide range of end points (Richter et al. 2007a; Welshons et al. 2006; Wetherill et al. 2007). These end points include those mediated by recently discovered estrogen response pathways initiated in human and animal cell membranes (nonclassical or alternative estrogen response mechanisms), which multiple laboratories have shown to be equally sensitive to BPA and estradiol in terms of activating effects in human and animal cells at low picomolar through low nanomolar concentrations (Alonso-Magdalena et al. 2008; Wetherill et al. 2007; Wozniak et al. 2005; Zsarnovszky et al. 2005).

The effects of BPA documented in these studies include a diverse array for which there are no data from GLP studies because the end points have not been examined: altered metabolism related to metabolic syndrome (Alonso-Magdalena et al. 2005, 2006, 2008; Ropero et al. 2008); altered adiponectin secretion (Hugo et al. 2008), which is a condition predicting heart disease and type 2 diabetes (Lang et al. 2008); altered epigenetic programming leading to precancerous lesions of the prostate (Ho et al. 2006); differential growth patterns in the developing prostate (Timms et al. 2005); abnormal growth, gene expression, and precancerous lesions of the mammary glands (Soto et al. 2008); and adverse effects on the female reproductive system, including uterine fibroids, para-ovarian cysts, and chromosomal abnormalities in oocytes (Newbold et al. 2007; Susiarjo et al. 2007). There is also a large literature on neuroanatomic, neurochemical, and behavioral abnormalities caused by low doses of BPA (Leranth et al. 2008; Richter et al. 2007a), which also are not capable of being detected by current GLP studies conducted for regulatory purposes because of their out-of-date assays.

The approaches used by academic and government scientists to study the potential health hazards of BPA contrast sharply with those still used by the chemical industry that are relied on by regulatory agencies in the United States and Europe, including the two studies identified by both the U.S. FDA and European Food Safety Authority (EFSA) as central to the decision to declare BPA safe at current human exposure levels (Tyl et al. 2002, 2008a). By using outdated and insensitive assays that were supposed to have been replaced by a new battery of screens and tests by 2000 [as mandated by the U.S. Congress in 1996 in the Food Quality Protection Act (1996), but which has, as yet, still not occurred], these studies conducted using GLP fail to find any adverse effects.

## Reliability and Validity

Reliability and validity are separate issues, although in the experimental research described here, validity and reliability basically refer to research that is credible. Golafshani (2003) noted that “reliability” refers to the extent to which results are consistent over time and are an accurate representation of the total population under study. Of central importance is that the results of a study must be reproduced under a similar methodology to be considered to be reliable. “Validity” refers to whether the research measures what it was intended to measure, and valid findings are considered to be true. In other words, reliability is determined by whether the results are replicable, whereas validity is assessed by whether the methods used result in finding the truth as a result of the investigator actually measuring what the study intended to measure.

## Use of GLP in Regulatory Decision Making

Despite strong evidence of aberrations caused by low doses of BPA in animals exposed during fetal and neonatal life in studies conducted by the world’s leading academic and government experts in the fields of endocrine disruption, endocrinology, neurobiology, reproductive biology, genetics, and metabolism, a relatively small number of studies reporting no adverse effects at low doses of BPA have continued to be promoted by the chemical industry and used by regulatory agencies (e.g., Ashby et al. 1999; Cagen et al. 1999; Tyl et al. 2002, 2008a). According to the U.S. FDA, these are accepted because they used GLP (U.S. EPA 2008), with the implication that studies not employing GLP are not reliable or valid (U.S. FDA 2008a).

### GLP does not guarantee reliability or validity of scientific results

Unfortunately, although GLP creates the semblance of reliable and valid science, it actually offers no such guarantee. GLP specifies nothing about the quality of the research design, the skills of the technicians, the sensitivity of the assays, or whether the methods employed are current or out-of-date. (All of the above are central issues in the review of a grant proposal by an NIH panel.) GLP simply indicates that the laboratory technicians/scientists performing experiments follow highly detailed U.S. EPA requirements [or in the EU, Organization for Economic Co-operation and Development (OECD) requirements] for record keeping, including details of the conduct of the experiment and archiving relevant biological and chemical materials (U.S. EPA 2008).

These record-keeping procedures in GLP were instituted because of widespread misconduct being committed by commercial testing laboratories (described above). These fraudulent results were possible because contract laboratory studies used in the regulatory process are rarely subject to the checks and balances that peer-reviewed, replicated scientific findings undergo. Without that acid test of reliability (replication by other independent scientists), other procedures were needed. Hence GLP was implemented, despite its severe limitations.

### NIH-funded research subject to more stringent reviews than GLP

Although few NIH-funded investigators adhere to GLP-mandated record keeping, the procedures of GLP are actually surpassed by the procedures required for NIH-funded science published in peer-reviewed journals. NIH-funded studies pass through three phases of peer review that are far more challenging than GLP requirements. First, the principal scientists must have demonstrated competence to conduct the research, and experimental methods, assays, and laboratory environment must involve use of state-of-the-art techniques to be competitive for NIH funding. Second, results are published in peer-reviewed journals, with detailed evaluations by independent experts examining all aspects of the study. And third, the findings are challenged by independent efforts to replicate; for example, the initial findings concerning the stimulating effects of estrogenic chemicals on the mouse prostate (Nagel et al. 1997; vom Saal et al. 1997) were independently replicated and extended by Gupta (2000), which led to an editorial identifying “initial results confirmed” (Sheehan 2000).

Typically, within a laboratory, interesting findings are also followed by subsequent publications extending the prior findings; examples include the findings of BPA effects on β cells in the mouse pancreas (Alonso-Magdalena et al. 2005, 2006, 2008) and the effects of estrogenic chemicals and drugs on the developing mouse prostate that followed earlier findings (described above) from this same group (Timms et al. 2005; Richter et al. 2007b). In particular, independent replication by competent, respected scientists is the main criterion of acceptance of the findings as having been demonstrated to be reliable and having been validated by virtue of coming to the same conclusion using a variety of sophisticated techniques in multiple publications.

An important criticism of the approach taken by the U.S. FDA in its assessment of the now approximately 1,000 articles on BPA is that it appears to have made no attempt to connect the dots between replicated studies; instead, the U.S. FDA appears to have assessed each study without regard to whether it had been confirmed by other studies.

Thus, collectively, many phases used to verify the reliability and validity of NIH-funded published research have been completely ignored by the U.S. FDA, whereas industry-funded GLP research is rarely, if ever, subject to these central requirements and yet is accepted by regulatory agencies as reliable and valid.

### The U.S. FDA’s misguided gold standard

In this light, the U.S. FDA’s reliance upon GLP as the gold standard is scientifically misguided. Furthermore, U.S. FDA administrators are ignoring published critiques of the GLP studies it considers reliable and valid, such as the study by Tyl et al. (2002) and two coordinated studies conducted at the same time by Ashby et al. (1999) and Cagen et al. (1999). Each was an industry-funded study conducted using GLP. Each was harshly criticized in peer-reviewed publications by academic scientists and government panels [Center for the Evaluation of Risks to Human Reproduction (CERHR) 2007; NTP 2001; vom Saal and Hughes 2005; vom Saal and Welshons 2006]. Yet, the U.S. FDA and EFSA panels still assert that these studies represent the gold standard in toxicologic research.

Specifically, the studies of Cagen et al. (1999) and Ashby et al. (1999) were recently rejected by the NTP CERHR panel on BPA as unusable for consideration in its evaluation of the health hazards posed by BPA (CERHR 2007). Both the Ashby et al. (1999) and Cagen et al. (1999) studies reported finding no effect of their positive control [the estrogenic drug diethylstilbestrol (DES)] on any outcome, although these failures were not acknowledged by the authors in either article. In experimental science, the failure of a positive control to show an effect indicates the experiment failed, which is the conclusion reached by the CERHR panel (CERHR 2007).

The Tyl et al. 2002 study, which the U.S. FDA still accepts as a major study for determination of the safety of BPA (U.S. FDA 2008a, 2008b), was criticized by an NTP panel that met in 2000 to examine the low-dose issue (NTP 2001), as well as in subsequent publications (vom Saal and Hughes 2005; vom Saal and Welshons 2006), for using an insensitive rat (the CD-SD rat) that requires extremely high doses (≥ 50 μg/kg/day) of the potent estrogenic drug ethinylestradiol to show effects such as those examined in the study by Tyl et al. (2002). This dose of ethinylestradiol is > 100 times higher than the approximately 0.3 μg/kg/day used by women in oral contraceptives. The fact that Tyl et al. (2002) adhered to GLP did not protect them from using insensitive animals. This led the NTP (2001) to state:

Because of clear species and strain differences in sensitivity, animal model selection should be based on responsiveness to endocrine-active agents of concern (i.e., responsive to positive controls), not on convenience and familiarity.

Thus, when reviewed by other scientists, three prior major GLP studies of BPA have been found to be so flawed as to be useless for guiding regulatory agencies in decision making. A new GLP study has now been published by Tyl et al. (2008a). Close examination of this study also reveals fatal flaws which render it useless for regulatory purposes, even though it conforms to GLP.

## Examples of Flaws Ignored by the U.S. FDA and EFSA in a Recent GLP Study of BPA

In summary, the flaws in Tyl et al. (2008a) are as follows:

- The high dose required for the positive control (estradiol) to cause an effect means the system used by Tyl et al. (2008a), at least in her laboratory, is relatively insensitive to exogenous estrogens and thus inappropriate for studying low-dose effects of estrogenic compounds such as BPA. The lack of response to low doses of estradiol or BPA in the Tyl laboratory is puzzling, in that the strain of mice used in these experiments (the CD-1 mouse) has been reported in > 20 other peer-reviewed publications to show adverse effects in response to very low doses of BPA (vom Saal 2008), as well as many other studies showing low-dose effects in response to the natural hormone estradiol, the estrogenic drugs ethinylestradiol and DES, and to other estrogenic chemicals.
- Tyl et al. (2008a) used insensitive, out-of-date protocols and assays that are incapable of finding many of the adverse effects reported by more sophisticated studies conducted by independent NIH-funded scientists as well as scientists funded by government agencies in other countries.
- In the specific case of testing for changes in prostate weight, Tyl et al. (2008a) reported an abnormally high prostate weight for control animals that exceeds by > 70% the prostate weights reported by other studies for animals of the same strain and similar age (e.g., Gupta 2000; Ruhlen et al. 2008). This suggests that the dissection procedures for the prostate in the Tyl laboratory included other nonprostatic tissues in the weight measurements, rendering them unusable for studying weight changes in the prostate in response to BPA or estradiol; neither chemical showed any effect on the selected end points, which directly contradicts other findings concerning opposite effects of low and high doses of estrogen on the prostate (Putz et al. 2001; Timms et al. 2005; vom Saal et al. 1997).

### Aberrant insensitivity of CD-1 mouse to estrogens

Tyl et al. (2008a) used estradiol as a positive control. It was fed to female mice before and during pregnancy and lactation at 80–220 μg/kg/day; after weaning, estradiol was fed to offspring at doses of 80–100 μg/kg/day. Estradiol was used as a positive control because BPA is a man-made endocrine-disrupting estrogenic chemical.

Many published findings reporting effects of very low doses of positive control estrogens and BPA in CD-1 mice demonstrate that the CD-1 mouse was somehow rendered insensitive in the test system used by Tyl et al. (2008a). The fact that a dose of 100–200 μg/ kg/day estradiol was necessary to show an effect of the positive control predicts that Tyl et al. (2008a) should not detect effects of BPA < 10–100 mg/kg/day, far above the low-dose range relevant to human exposures that was supposedly of interest.

For nuclear estrogen receptor–mediated effects via regulation of gene activity (nuclear estrogen receptors are transcription factors whose activity is regulated by binding to estrogen), prior studies have typically shown a 1,000-fold lower activity for BPA relative to estradiol or potent estrogenic drugs, including DES and ethinylestradiol. For example, Richter et al. (2007b) reported an increase in androgen receptor gene activity to estradiol at 1 pM (0.28 pg/mL) in fetal CD-1 mouse prostatic mesenchyme cells in primary culture, and the same response was found for BPA at 1,000 pM (228 pg/mL); the *in vitro* response to estradiol was predicted by the response of the prostate to increasing free serum estradiol from 0.2 to 0.3 pg/mL in male mouse fetuses via estradiol administration to the mother (vom Saal et al. 1997). Other research showed that a significant effect on development of the male reproductive system in CF-1 mice occurred at a maternal dose of 0.002 μg/kg/day ethinylestradiol (Thayer et al. 2001), similar to effects observed with 2–20 μg/kg/day BPA (vom Saal et al. 1998). The research of Honma et al. (2002) showed accelerated puberty in CD-1 (ICR) mice at a DES dose of 0.02 μg/ kg/day (the positive control), and the same response to BPA occurred at 20 μg/kg/day, again revealing a 1,000-fold difference between the positive control estrogen and BPA.

There are many other examples of findings where a higher dose of BPA was required to cause the same effect as the positive control estrogen (estradiol, ethinylestradiol, or DES) in studies where the effects were mediated by the classical nuclear estrogen receptors, in contrast to the more recently discovered rapid signaling estrogen response system where BPA and these positive control estrogens have equal potency, as described above. In summary, CD-1 mice have been used by a large number of academic and government investigators and have been reported in peer-reviewed publications to be sensitive to positive control estrogens within the range of human sensitivity based on *in vivo* and *in vitro* studies via the classical estrogen receptor α–mediated response mechanism. The CD-1 mouse is the animal model that has been used by the U.S. National Institute of Environmental Health Sciences (NIEHS) for decades, because it is considered the best animal model for predicting the effects of developmental exposure to estrogen in humans (Newbold 1995; Newbold et al. 2007).

The failure of traditional toxicologic studies conducted by Tyl et al. (2008a, 2008b) to detect the wide range of adverse effects of even relatively high doses of BPA or of low doses of estradiol that have been reported in numerous studies by academic and government scientists provides evidence that the GLP protocols established long ago by regulatory agencies to determine the toxicity of chemicals are inappropriate for detecting the endocrine-disrupting activities of chemicals such as BPA. Indeed, this was the premise of the congressional mandate in the Food Quality Protection Act (1996) for the U.S. EPA to establish a new set of assays for endocrine-disrupting chemicals, although this process has been systematically delayed and is > 8 years behind the congressionally mandated date of 2000 to have these new assays validated.

Citing Tyl et al. (2008a), the EFSA report on BPA (EFSA 2006) stated that “the positive control substance, 17β-estradiol, resulted in reproductive and developmental toxicity.” This report failed to acknowledge that only a very high dose of the positive control was sufficient to elicit effects and that this meant that the experiments conducted in the Tyl laboratory were for some reason very insensitive to any estrogen and thus inappropriate for use in a study to examine low-dose estrogenic effects of BPA.

Based on the preliminary report released by the U.S. FDA regarding BPA (U.S. FDA 2008a), it appears that the U.S. FDA has followed the lead of the EFSA in its lack of understanding of the importance of the dose of the positive control estrogen required to cause adverse effects. The consequence is that the U.S. FDA has relied primarily on the study of Tyl et al. (2008a, 2008b), with the result that the U.S. FDA has assured Americans that BPA is safe at current human exposure levels.

Several factors might account for the insensitivity of the CD-1 mouse in the Tyl et al. studies (2008a, 2008b) conducted at Research Triangle Institute (RTI), a testing facility that conducted these (as well as previous) studies funded by the American Chemistry Council. One possibility is that the diet used in these studies may have interfered with the results. The feed used by Tyl et al. (2008a) in this experiment (Purina 5002) has been shown by others to interfere with responses to exogenous estrogenic chemicals, blocking adverse effects documented on other diets. For example, a number of years ago, Thigpen et al. (2003) at the NIEHS recommended against the use of Purina 5002 in studies of endocrine-disrupting chemicals. Tyl et al. (2008a) measured some specific phytoestrogens in Purina 5002 feed by chemical analysis; however, in a report on NIH-sponsored meetings on this subject, Heindel and vom Saal (2008) pointed out that this is an insufficient control for total dietary estrogenic contaminants that can disrupt studies involving the effects of estrogenic chemicals.

A second possibility is that there are strain differences in sensitivity developed in the CD-1 mouse sold by the various Charles River Laboratories located in different regions. We consider this unlikely, because most laboratories regularly replace their CD-1 mouse breeder stock from Charles River Laboratories, and practices there make it unlikely that the sensitivity of this outbred stock to estrogens has changed dramatically over a very short period of time. Also, because RTI, where the Tyl studies were conducted, is very near the laboratories of the NIEHS, it is likely that the CD-1 mice used by these two programs were purchased from the same breeding facility.

### Use of insensitive, out-of-date protocols and assays

Another serious concern about the two recent studies by Tyl et al. (2008a, 2008b) is the experimental approach used, thus raising questions about the validity of the studies. The study design used by Tyl et al. (2008a, 2008b) has been superseded by advances in both experimental design and analytical tools developed by NIH-funded scientists (and their counterparts in Europe and Asia) since the mid-1990s. The methods used by Tyl et al., primarily wet weight changes of tissues, gross histologic changes, and developmental landmarks such as vaginal opening, were established procedures by the 1950s. Thus, a major limitation of the Tyl studies is the failure to measure more meaningful and sensitive end points in order to detect the effects of low-dose BPA exposure, which are often not macroscopic in nature. Indeed, in 2001, the director of the reproductive division of the National Health and Environmental Effects Research Laboratory at the U.S. EPA stated that the inconclusive results concerning effects of BPA on reproductive toxicology can only be solved by understanding the mechanisms (Triendl 2001). With current GLP standards it is not possible to study mechanisms because they still rely on out-of-date assays.

As one example of a comparison between the approach by Tyl et al. (2008a) and independent government-funded academic scientists, extensive research has been conducted by Soto et al. (2008) and by other independent academic and government scientists describing effects of exposure of female mice and rats to very low doses of BPA during peri-natal development on the mammary glands (Jenkins et al. 2009). Although Tyl et al. (2008a) reported no low-dose effects of BPA on the mammary glands using conventional histologic analysis, there have been consistent findings of adverse effects of low doses of BPA from studies that used more sophisticated and sensitive analysis of whole mounted mammary glands to facilitate detection of microscopic lesions, coupled with immunostaining for regulatory proteins as well as techniques for determination of aberrant gene expression associated with progression to cancer. These peer-reviewed studies have reported detecting changes during embryonic development of mammary glands as well as abnormalities detected during adolescence through adulthood that are indicative of mammary gland cancer as well as other developmental abnormalities (Colerangle and Roy 1997; Durando et al. 2007; Jenkins et al. 2009; LaPensee et al. 2008; Markey et al. 2001, 2005; Moral et al. 2008; Munoz-de-Toro et al. 2005; Murray et al. 2007; Nikaido et al. 2004; Vandenberg et al. 2006, 2007b; Wadia et al. 2007).

Similar to the findings for the mammary gland, Ogura et al. (2007) reported that if tissues were analyzed by conventional histologic methods (staining with hematoxalin and eosin), prenatal exposure to low doses of BPA or DES showed no effects on prostate development, whereas if the sections were analyzed using antibodies that identified basal cells and basal cell squamous metaplasia, then significant effects were revealed. Squamous metaplasia of basal cells indicates abnormal proliferation and function of the prostate stem cell population that is thought to transform into neoplastic cells; Ho et al. (2006) reported that neonatal exposure to very low doses of BPA caused 100% of male rats to develop high-grade prostatic intraepithelial neoplastic lesions later in life. All of these studies were rejected by the U.S. FDA as not adequate for making regulatory decisions about the safety of BPA. Instead, the U.S. FDA relied upon Tyl et al. (2008a), even though the study used techniques that Ogura et al. (2007) showed lacked the sensitivity of 21st century experimental approaches.

Although findings regarding changes in brain structure, brain chemistry, and behavior represent the largest portion of the literature on low-dose BPA, Tyl et al. (2008a) did not examine any neurobehavioral end points. The NTP (2008) and the NIEHS conference consensus reports (vom Saal et al. 2007) both indicated concern about neurobehavioral effects of low doses of BPA. Thus, the absence of studies that included neurobehavioral end points is a glaring omission of Tyl et al. (2008a, 2008b).

### Flawed prostate dissection

Data presented by Tyl et al. (2008a) raise questions about the adequacy of techniques used in their BPA studies. Specifically, Tyl et al. (2008a) reported that the prostate in 3.5-month-old control male CD-1 mice weighed > 70 mg [see Table 3 in Tyl et al. (2008a) for data on F_1_ retained males]. This average control weight contrasts sharply with those reported from other laboratories. Specifically, the weight of the prostate in 2- to 3-month-old CD-1 mice using the dissection technique based on both Ruhlen et al. (2008) and Gupta (2000) and at the NIEHS (Newbold RR, personal communication) is about 40 mg. Several studies have reported that prenatal exposure to very low doses of BPA and positive control estrogens increased prostate size, prostatic androgen receptors, and prostate androgen receptor gene activity (Gupta 2000; Richter et al. 2007b; Thayer et al. 2001; Timms et al. 2005; vom Saal et al. 1997), but the enlarged prostate of experimental animals exposed to BPA in these laboratories weighed less than the prostates in the control animals of Tyl et al. (2008a). This raises serious questions about the procedures and/or animals used by Tyl et al. The weight of prostate reported by Tyl et al. (2008a) suggests that the technique used for dissecting the prostate resulted in non-prostatic tissue being weighed along with prostate. The seminal vesicle, coagulating gland, and dorsolateral prostate all merge together where the ejaculatory ducts enter the urethra, and there are also fat deposits on the prostate. This poses a challenge for those without proper training in distinguishing these different tissues during dissection in mice.

Alternatively, as male rodents age, they are prone to develop prostatitis. Although this inflammatory disease leads to an increase in prostate size and could thus account for the very large prostate weights reported by Tyl et al. (2008a), anyone familiar with the appearance of prostatitis would detect this abnormality upon histologic examination, which Tyl et al (2008a) supposedly conducted. Also, prostatitis is rare in young-adult mice or rats (Cowin et al. 2008), and the size of the prostates in the Tyl et al. (2008a) study were similar to those for middle-aged and old male mice.

The findings regarding effects of BPA on the prostate presented by Tyl et al. (2008a) are thus suspect and cannot be used as evidence that other earlier studies (Gupta 2000; Timms et al. 2005; vom Saal et al. 1997) are not replicable. Given these problems in prostate weight measurements, it is not surprising that even very high doses of BPA or estradiol reported by Tyl et al. (2008a) had no effect on the prostate, in sharp contrast to other studies that showed stimulation of the prostate at low doses of estrogen and inhibition at high doses (Putz et al. 2001; Timms et al. 2005).

In addition to the problem associated with the high prostate weight reported by Tyl et al. (2008a), in a separate measurement the authors combined the anterior prostate (coagulating gland) and seminal vesicle, presenting these two organs as one combined outcome measure. This is wrong and misleading. The coagulating glands emerge as the anterior ducts of the prostate from the dorsocranial region of the urogenital sinus, whereas the seminal vesicles bud from the proximal region of the Wolffian ducts. Elevated estrogen is associated with an increase in prostate size associated with an increase in prostate androgen receptors, whereas a decrease in seminal vesicle size is associated with a reduction in 5α-reductase, an enzyme that converts testosterone to the more potent androgen 5α-dihydrotestosterone (Nonneman et al. 1992). Low doses of BPA have been shown to decrease the size of organs that differentiate from the embryonic Wolffian ducts (epididymides and seminal vesicles) while increasing the size of regions of the prostate that develop from the urogenital sinus (vom Saal et al. 1998). Combining these different organs (it is technically not difficult to separate them) was thus inappropriate because they develop from different embryonic tissues that show markedly different responses to estrogenic chemicals during development. In fact, Ogura et al. (2007) reported that the anterior prostate (coagulating glands) showed the greatest expression of ER-α, and also showed the most pronounced indication of basal cell squamous metaplasia in response to developmental exposure to low doses of DES and BPA relative to other regions of the prostate.

## Conclusions

Because the control data of Tyl et al. (2008a) were not consistent with the prior published literature for prostate weight of young-adult CD-1 male mice and because their methods were inappropriate for revealing an extensive body of adverse effects detected using more sophisticated approaches, we deem the findings by Tyl et al. to be invalid. Hundreds of studies show adverse effects of BPA in animals, with many conducted at concentrations equivalent to current human levels of BPA exposure; thus, it is unlikely that academic scientists would bother to replicate the outdated approaches used by Tyl et al. (2008a, 2008b). This lack of replication is typical of GLP studies, which tend to involve unnecessarily large numbers of animals [Tyl et al. (2002) used > 8,000 rats], and reliability appears to be accepted because of the numbers of animals that were used. Although using excessive numbers of animals is accepted as good science by the U.S. FDA, the use of arbitrarily large numbers of animals per group (> 20 animals per treatment group is common) actually violates guidelines in the NIH *Guide for the Care and Use of Laboratory Animals* (Institute of Laboratory Animal Research 1996) that govern research conducted by academic and government scientists. For research with animals to be approved by any university animal care and use committee, group sizes must be based on power analysis conducted using historic data. Based on this criterion in the NIH Guide, all of the studies by Tyl et al. were significantly over powered and thus in direct violation of federal guidelines for conducting animal research, a fact about which U.S. FDA regulators seem unaware.

Each of the four main industry-funded GLP studies of BPA (Ashby et al. 1999; Cagen et al. 1999; Tyl et al. 2008a, 2008b) is flawed and not appropriate for use in setting health standards. Clearly, meeting GLP standards is not a guarantee of reliable or valid science. It is of great concern that the U.S. and EU regulatory communities are willing to accept these industry-funded, antiquated, and flawed studies as proof of the safety of BPA while rejecting as invalid for regulatory purposes the findings from a very large number of academic and government investigators using 21st-century scientific approaches. The basis for these decisions by U.S. and EU regu latory agencies should be thoroughly investigated, particularly since the NTP (2008) concluded that BPA exposure to human infants was in the range shown to cause harm in experimental animals and since both the Canadian Ministry of Health and the Ministry of the Environment recently concluded that BPA was a toxic chemical (Environment Canada 2008).

Problems inherent with reliance on GLP as the standard for choosing data are compounded by the process used by federal agencies to determine membership on science advisory panels. Leading experts qualified by specific experience on the chemical or end points under consideration are often specifically excluded from membership. For example, the U.S. FDA’s BPA review panel was identified as an expert panel, when in fact the panel was composed largely of scientists lacking any experience in research with BPA. This process, which appears to consider almost any scientist knowledgeable about a chemical to create bias, makes it vastly more difficult for the panel to integrate scientific data from the relevant literature, especially since, as with BPA, there are almost 1,000 relevant studies and the review panel is provided with very little time to become knowledgeable about the details. It means that the depth of knowledge present on this and similarly constituted government regulatory agency panels is unlikely to be sufficient to subject draft assessments to the scrutiny that peer review by experts normally entails. Combined with reliance on GLP data, this process has a high potential to yield flawed assessments that jeopardize public health.

We are not suggesting that GLP should be abandoned as a requirement for industry-funded studies. We object, however, to regulatory agencies implying that GLP indicates that industry-funded GLP research is somehow superior to NIH-funded studies that are not conducted using GLP. This argument demonstrates a lack of understanding of the profound difference between the use of replication as a mechanism to assess reliability and the methods used to assess validity for peer-reviewed published academic studies, whereas GLP was instituted with the expectation that this type of verification would not occur.

Public health decisions should be based on studies using appropriate protocols and the most sensitive assays. They should not be based on criteria that include or exclude data depending on whether or not the studies use GLP. Simply meeting GLP requirements is insufficient to guarantee scientific reliability and validity.
